# Effects of Mechanical Stretch on Cell Proliferation and Matrix Formation of Mesenchymal Stem Cell and Anterior Cruciate Ligament Fibroblast

**DOI:** 10.1155/2016/9842075

**Published:** 2016-07-25

**Authors:** Liguo Sun, Ling Qu, Rui Zhu, Hongguo Li, Yingsen Xue, Xincheng Liu, Jiabing Fan, Hongbin Fan

**Affiliations:** ^1^Department of Orthopedic Surgery, Xijing Hospital, The Fourth Military Medical University, Xi'an 710032, China; ^2^Tianjin Sanatorium, Beijing Military Region, Tianjin 300381, China; ^3^Department of Clinical Laboratory, Xijing Hospital, The Fourth Military Medical University, Xi'an 710032, China; ^4^College of Science, Air Force Engineering University, Xi'an 710051, China; ^5^Division of Advanced Prosthodontics, School of Dentistry, University of California, Los Angeles, CA 90095, USA

## Abstract

Mesenchymal stem cells (MSCs) and fibroblasts are two major seed cells for ligament tissue engineering. To understand the effects of mechanical stimulation on these cells and to develop effective approaches for cell therapy, it is necessary to investigate the biological effects of various mechanical loading conditions on cells. In this study, fibroblasts and MSCs were tested and compared under a novel Uniflex/Bioflex culture system that might mimic mechanical strain in ligament tissue. The cells were uniaxially or radially stretched with different strains (5%, 10%, and 15%) at 0.1, 0.5, and 1.0 Hz. The cell proliferation and collagen production were compared to find the optimal parameters. The results indicated that uniaxial stretch (15% at 0.5 Hz; 10% at 1.0 Hz) showed positive effects on fibroblast. The uniaxial strains (5%, 10%, and 15%) at 0.5 Hz and 10% strain at 1.0 Hz were favorable for MSCs. Radial strain did not have significant effect on fibroblast. On the contrary, the radial strains (5%, 10%, and 15%) at 0.1 Hz had positive effects on MSCs. This study suggested that fibroblasts and MSCs had their own appropriate mechanical stimulatory parameters. These specific parameters potentially provide fundamental knowledge for future cell-based ligament regeneration.

## 1. Introduction

Anterior cruciate ligament (ACL) is an important intra-articular structure to maintain the stability of knee joint. However, it cannot heal spontaneously after severe injury due to poor vascularization [1–3]. Allografts or autografts (hamstring or patella tendon) are now frequently used to reconstruct ACL because of the poor results of synthetic grafts. Although the promising results such as subjective satisfaction and partial stability restoration are acquired by allo/auto graft transplantation, no reliable and functional tissue repair is achieved in long-term follow-up studies. The increased concerns including ligament laxity, donor site morbidity, and pathogen transfer are observed in clinical treatments [4–6]. Recently tissue-engineered ligament provides a new approach to the solution of aforementioned problems.

Tissue-engineered ligament has the potential to provide an alternative graft that could be readily available. However, construction of a viable and biomechanically equivalent ligament requires a fundamental understanding of ACL biology including fibroblast matrix synthesis and remodeling in response to the local mechanical environment [7]. The properties of ligament including structure, function, heal capability, and development are significantly affected by mechanical stimulus. With daily activities, the ACL is subjected to varying amounts of tensile strain, which is crucial for ligament homeostasis. Mechanical loads induce changes in the structure, composition, and function of living tissues. It is now well recognized that mechanical forces play a fundamental role in the regulation of cell functions, including gene induction, protein synthesis, cell growth, death, and differentiation, which are essential to maintain tissue homeostasis [8]. Another study also showed that mechanical loads affect cellular functions such as cell proliferation and collagen synthesis [9].

To reconstruct a functional tissue-engineered ligament, selection of cell source is of great importance. Due to differences in phenotype and function, different seed cell will greatly influence the properties of tissue-engineered ligament. ACL fibroblasts are load-sensitive cells and their complex structure changes in response to mechanical forces. Furthermore, the collagen produced by fibroblasts is the main component of ligament and has great tensile strength [10]. Theoretically, ACL fibroblast should be the primary choice for potential ligament tissue engineering, because especially they could be easily harvested in diagnostic arthroscopy procedure. In addition to ACL fibroblasts, mesenchymal stem cell (MSC) isolated from bone marrow is another potential cell source for ligament repair due to their multipotent and proliferate capabilities. The scaffold fabricated from woven silk fibers has mechanical properties similar to the native ACL, showing the abilities to enhance MSCs attachment, proliferation, and differentiation [11]. To potentially improve the functionality and structure of tissue-engineered ligament, fibroblasts forming ACL and medial collateral ligament (MCL) tissues were compared with MSCs in previous studies. The proliferation rate and collagen excretion of MSCs were further shown to be higher than ACL and MCL fibroblasts [12]. Although many studies investigated the influence of cyclic mechanical stimulation on graft incorporation, cell morphology, collagen production, and cellular differentiation, few literatures have characterized the optimal parameter of mechanical stimulation [13–15].

In an effort to better understand the effects of mechanical stimulation on different cells and to develop effective approaches for cell therapy, it is necessary to study the biological effects of various mechanical loading conditions on cells. In this study, fibroblasts and MSCs were tested and compared under a novel Uniflex/Bioflex culture system that may mimic mechanical strain in ligament tissue. The objective is to find the optimal parameters (magnitude, frequency, and duration of strain) required for cell proliferation and collagen production, which potentially provides fundamental knowledge for future cell-based ligament regeneration.

## 2. Materials and Methods

### 2.1. Isolation and Expansion of MSC and Fibroblast

MSCs and fibroblasts were, respectively, isolated from bone marrow aspirates and ligament tissues of New Zealand White rabbits (12 weeks old, 2.5–3.0 kg) following the methods previously reported [16]. In general, mononuclear cells from bone marrow were separated by centrifugation in a Ficoll-Hypaque gradient (Sigma Co., St. Louis) and suspended in 20 mL of Dulbecco's Modified Eagle Medium (DMEM) supplemented with 10% fetal bovine serum (FBS) (HyClone Logan, Utah), l-glutamine (580 mg/L), and penicillin-streptomycin (100 U/mL). Cultures were incubated at 37°C and 5% CO_2_. After 72 h, nonadherent cells were removed by changing medium. When reaching 70–80% confluence, adherent cells were freed from the flask with 0.05% trypsin and subcultured. A homogenous MSCs population was obtained after 2 weeks of culture and MSCs (passage 3) were harvested for further use.

For fibroblasts isolation, the collected rabbit ACL was excised under sterile condition. The ligament tissue was minced and washed twice in 1% antibiotic medium for 10 min. The minced ligament tissue was then placed in a solution of 0.25% collagenase at 37°C and agitated overnight for 12–18 h. Fibroblasts were isolated by straining the digest through a 100 *μ*m filter. The cell-containing solution was centrifuged at 300 g for 5 min, the supernatant removed, and the pellet resuspended in 1% antibiotic medium and recentrifuged. The supernatant was removed and the cells suspended in culture medium with 1% antibiotic, 1% glutamine, and 10% fetal bovine serum (FBS) and cultured in T-75 flasks at 37°C, 100% humidity, and 5% CO_2_. Confluence was achieved in 2 weeks and subculture was performed. The fibroblasts (passage 3) were collected for further evaluation.

### 2.2. Cell Culture in Uniflex/Bioflex Plate

Cells were trypsinized by adding 1 mL of 0.25% trypsin solution to a T75 flask with confluent cells followed by 3 min incubation at 37°C with regular gentle shaking. The trypsin reaction was stopped by adding 10 mL of culture medium containing 10% FBS. The cell suspension was then centrifuged at 300 g for 10 min at 20°C. The cell pellet was resuspended in 2 mL of medium (1% antibiotic, 1% glutamine, and 10% FBS) and thoroughly mixed by repeated pipetting. 1 × 10^6^ cells were seeded in each well of the Uniflex/Bioflex culture plates and incubated at 37°C, 100% humidity, and 5% CO_2_.

### 2.3. Mechanical Loading

#### 2.3.1. Uniaxial Strain

**Figure 1:**
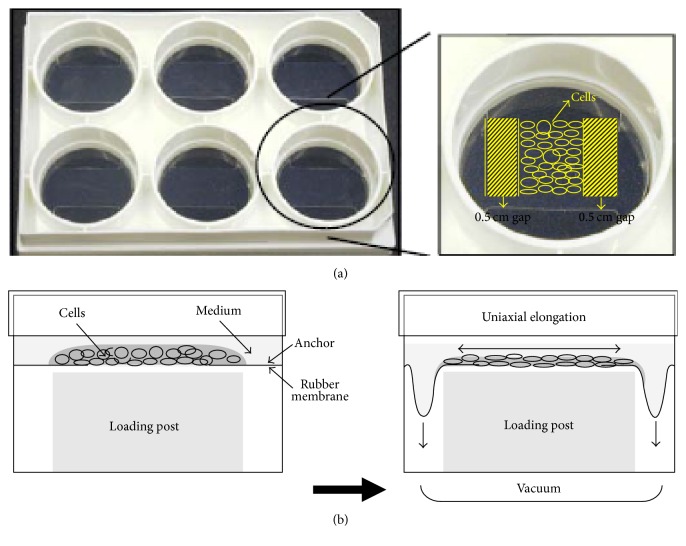
(a) Formation of cell sheet construct on Uniflex culture plate; (b) diagram of side view of uniaxial stretch system.

The fibroblasts and MSCs were, respectively, loaded in each well of Uniflex culture plates at 37°C, 100% humidity, and 5% CO_2_ until it reached confluence. A 0.5 cm gap was made on each side of the cell seeding area for allowing cell migration and proliferation (Figure 1(a)). The cells were uniaxially loaded by placing loading rectangle posts (Flexcell International) beneath each well of the Uniflex culture plates in a gasketed baseplate and applying vacuum to deform the flexible membranes downward. The flexible membrane deformed downward along the long sides of the loading posts thus applying uniaxial strain to loaded cells (Figure 1(b)). The loading regimen was for 5 days, 8 h/day (with 15 min rest every 2 h) at 5, 10, and 15% strain and 0.1, 0.5, and 1 Hz, using a Flexcell Strain Unit (Flexcell International).

#### 2.3.2. Radial Strain

**Figure 2:**
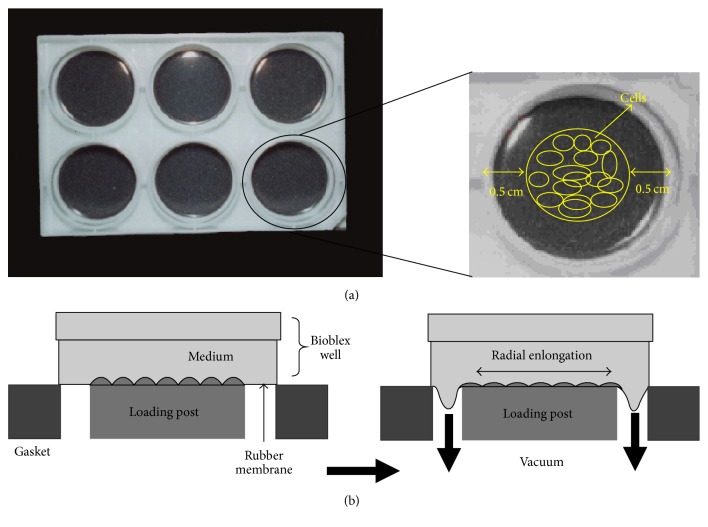
(a) Formation of cell sheet construct on Bioflex culture plate; (b) diagram of side view of radial stretch system.

The fibroblasts and MSCs from T75 flask were trypsinized and cultured in medium (1% antibiotic, 1% glutamine, and 10% FBS) in each well of Bioflex culture plates at 37°C, 100% humidity, and 5% CO_2_ until it reached confluence. A 0.5 cm gap was made around the cell seeding area allowing space for cell migration and proliferation (Figure 2(a)). The cells were radially loaded by placing cylindrical loading posts (Flexcell International) beneath each well of the Bioflex culture plates in a gasketed baseplate and applying vacuum to deform the flexible membranes downward. The flexible membrane deformed downward along the circumference of the cylindrical loading posts thus applying radial strain to ACL fibroblast (Figure 2(b)). The loading regimen was for 5 days, 8 h/day (with 15 min rest every 2 h) at 5, 10, or 15% strain and 0.1, 0.5, or 1 Hz, using a Flexcell Strain Unit (Flexcell International).

### 2.4. Cell Viability/Proliferation

Alamar Blue (AB, Sacramento. CA) was added into the culture media in the 6-well plate at a final concentration of 10% and was incubated for 2 h at 37°C (AB mixture should turn to a purplish/reddish shade). After incubation for 2 h, triplicates of 100 *μ*L AB mixture from each well were transferred and placed in a 96-well plate. Optical density of the AB mixture was measured at 570 and 600 nm with a standard spectrophotometer.

The oxidized form of AB is nonfluorescent and blue (*λ*_max_ = 600 nm), whereas the reduced form is fluorescent and red (*λ*_max_ = 570 nm). The proposed mechanism by which the dye detects living cells involves metabolic-based reduction via reactions of the respirator chain. The number of viable cells correlates with the magnitude of dye reduction and is expressed as percentage of AB reduction [17]. The percentage of AB reduction (% AB reduction) was calculated according to the manufacturer's protocol. It was corrected for background values of negative controls containing medium without cells.

### 2.5. Collagen Production Assay

The culture medium was completely removed from the 6-well plates. The seeded cells were washed twice with PBS solution. The pepsin (0.025%) was then added to the wells and incubated with cells for 2 h to digest all synthesized collagen. The solubilized collagen was neutralized with 1 M NaOH and aliquot to microcentrifuge tubes. 300 *μ*L of Sircol Dye reagent was added to 100 *μ*L of solubilized collagen and was shaken for 30 min. During this period the Sircol Dye will bind to soluble collagen. The dye reagent is designed so that the collagen-dye complex will precipitate out of solution. The microcentrifuge tubes were spun at 10,000 ×g for a 10 min. It is important to firmly pack the insoluble pellet of the collagen-dye complex at the bottom of the tubes, so as to avoid any loss during draining. The unbound dye solution is removed by carefully inverting and draining the tubes. Alkali Reagent (500 *μ*L) was added to each tube and vortexed to release the bound dye into solution. 150 *μ*L aliquots of the released bound dye were transferred into a microtitter plate. The absorbance was read at 540 nm and reference wavelength at 600 nm.

### 2.6. Statistical Analysis

Unpaired* t*-test was used for statistical data analysis of the stretching effects on cells at a significance level of 0.05 and sample size of 6.

## 3. Results

### 3.1. Uniaxial Stretch for Fibroblasts

After 5%, 10%, and 15% stretching at 0.1 Hz, 8 hrs/day for 5 days, the ACL fibroblast proliferation decreased significantly by 3.9%, 4.1%, and 13.1%, respectively (*p* < 0.05). The collagen production was decreased significantly by 21%, 14%, and 11.1%, respectively. (*p* < 0.05).

5% and 15% stretching of the ACL fibroblast at 0.5 Hz significantly increased cell proliferation by 6% and 11%, respectively (*p* < 0.05). 10% stretch at 0.5 Hz significantly decreased cell proliferation by 5% (*p* < 0.05). Collagen production was significantly decreased by 15.1% when the cells are stretched at 5% and 0.5 Hz (*p* < 0.05). However, when the cells are stretched at 10% and 15% with the same frequency, collagen production was increased by 3.0% (*p* < 0.05) and 33.9% (*p* < 0.05), respectively.

**Figure 3:**
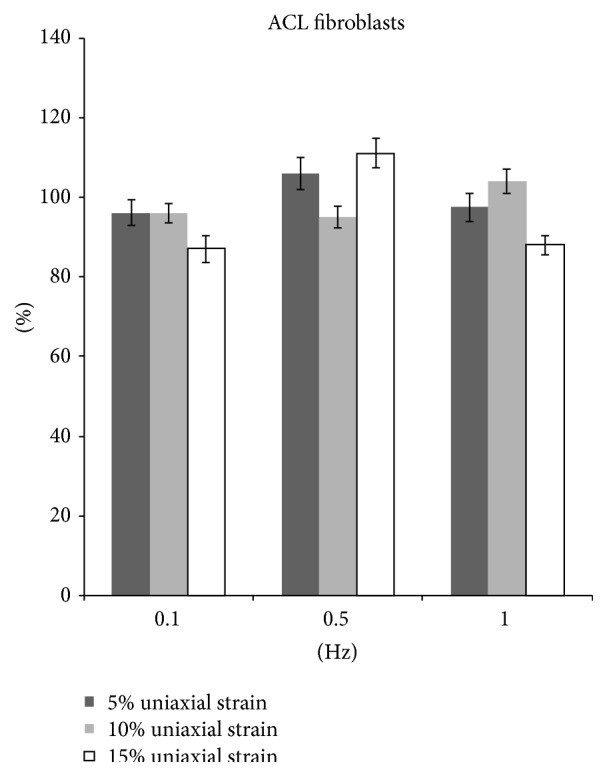
The proliferation of fibroblasts after uniaxial stretch stimulation.

**Figure 4:**
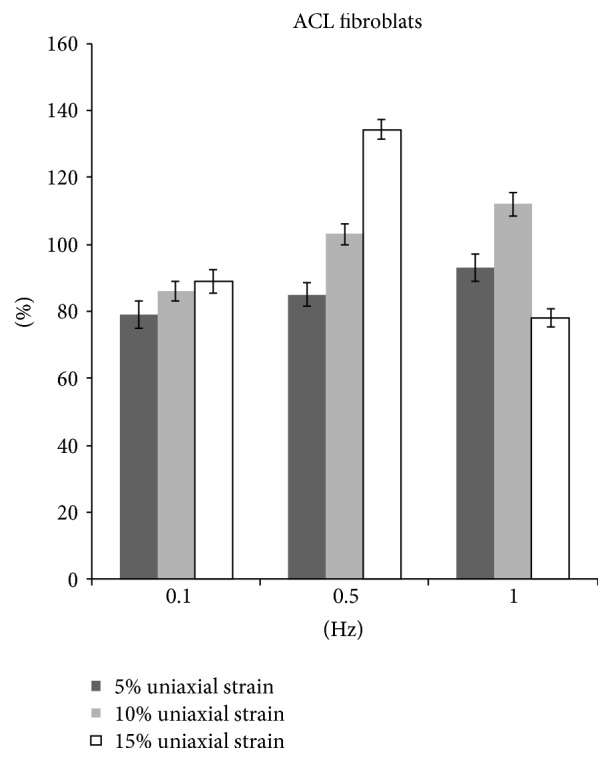
The collagen production of fibroblasts after uniaxial stretch stimulation.

Cyclic stretching of ACL fibroblast at 1 Hz with magnitude of either 5% or 15% showed a decrease in cell proliferation by 2.5% and 12.0%, respectively (*p* < 0.05). Similarly, the collagen production was decreased by 7.0% and 21.9%, respectively (*p* < 0.05). On the other hand, 10% stretch at 1 Hz increased cell proliferation by 4.0% (*p* < 0.05) and collagen production by 12% (*p* < 0.05) (Figures 3 and 4).

### 3.2. Uniaxial Stretch for MSCs

**Figure 5:**
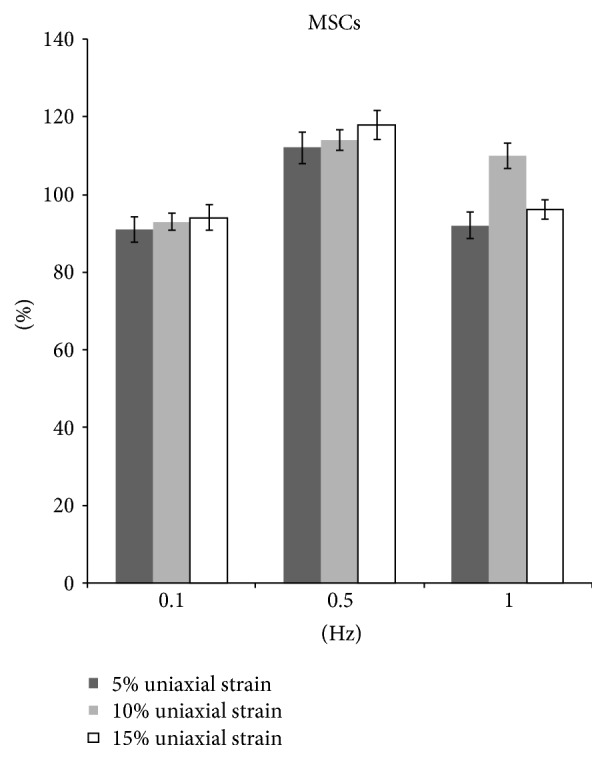
The proliferation of MSCs after uniaxial stretch stimulation.

The proliferation of MSCs showed similar trend with fibroblasts. After 5%, 10%, and 15% stretching at 0.1 Hz, 8 hrs/day for 5 days, the MSCs proliferation all decreased significantly (*p* < 0.05). However, when stretching at 0.5 Hz with 5%, 10%, and 15% strain, the proliferation all increased by 12%, 14%, and 18% (*p* < 0.05). When the frequency increased to 1 Hz, only 10% strain could enhance proliferation (Figure 5).

**Figure 6:**
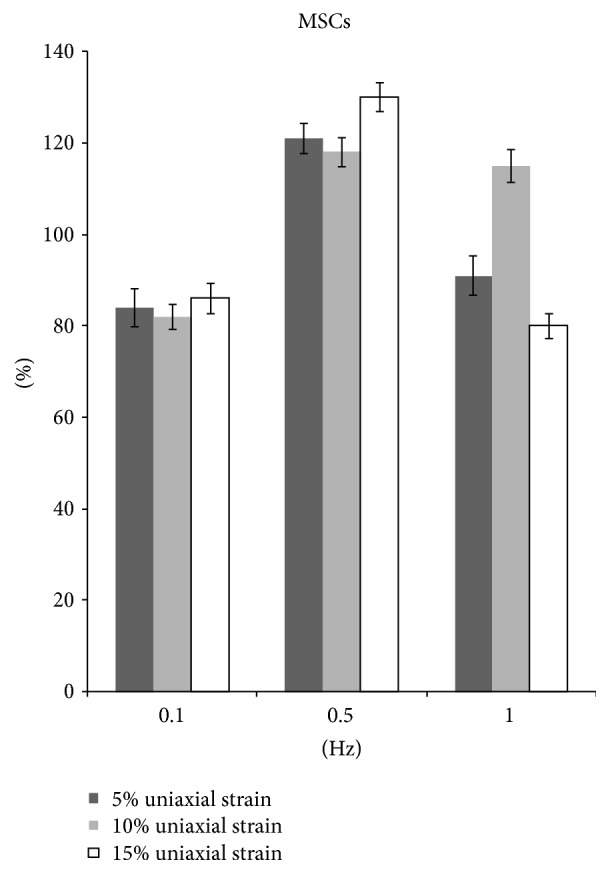
The collagen production of MSCs after uniaxial stretch stimulation.

MSCs showed the decreased collagen production at 0.1 Hz with magnitude of either 5%, 10%, or 15% (*p* < 0.05). On the contrary, the collagen production increased by 21%, 18%, and 30%, respectively, at 0.5 Hz with 5%, 10%, and 15% strain (*p* < 0.05). At 1 Hz, only 10% stretch increased collagen production by 15% (*p* < 0.05) (Figure 6).

### 3.3. Radial Stretch for Fibroblasts

**Figure 7:**
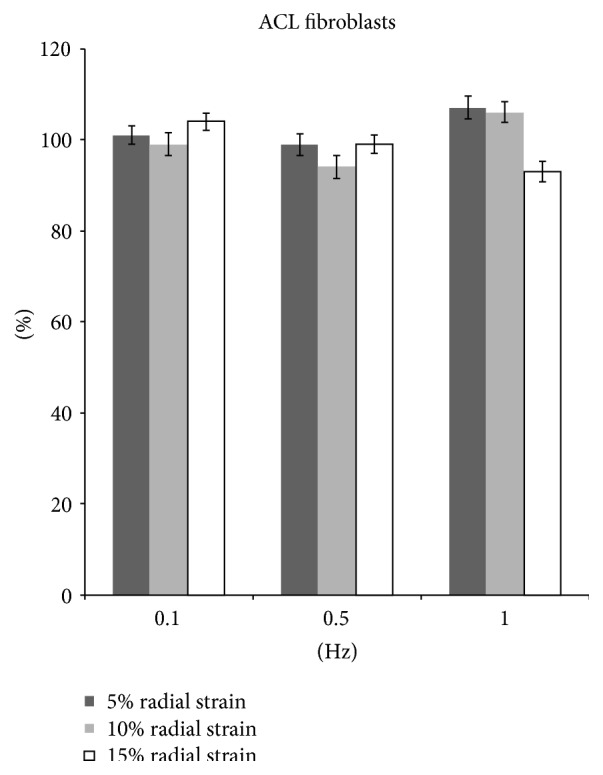
The proliferation of fibroblasts after radial stretch stimulation.

**Figure 8:**
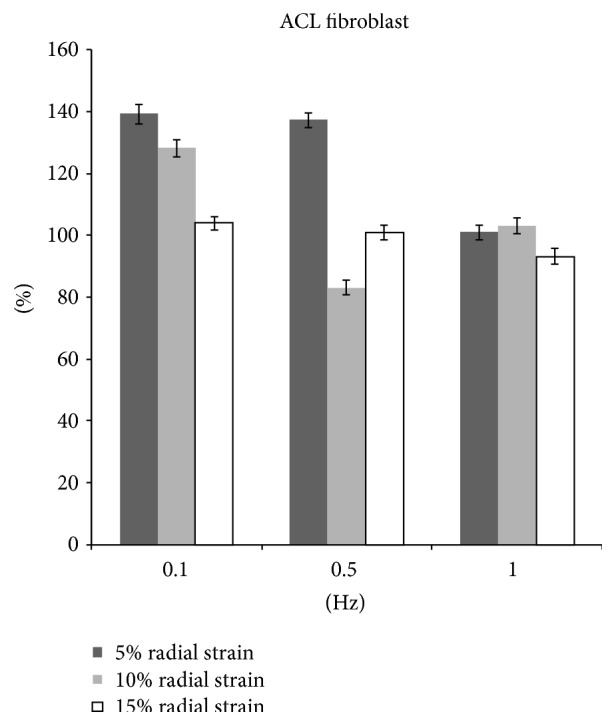
The collagen production of fibroblasts after radial stretch stimulation.

After 15% stretching at 0.1 Hz, 8 hrs/day for 5 days, the ACL fibroblast proliferation increased by 4% (*p* < 0.05). No significant difference was detected in cells with 5% and 10% stretching as compared to unstretched cells (Figure 7). However, there was a significantly increased collagen production by 39.3%, 28.1%, and 4.0% in 5%, 10%, and 15% radial strain groups, respectively (Figure 8).

At 0.5 Hz, 10% stretch group showed a decrease in proliferation by 6.0% (*p* < 0.05) and collagen production by 17.0% (*p* < 0.05). In 5% stretch group, an increase in collagen production by 37.2% (*p* < 0.05) was observed although the cell proliferation showed no significant difference compared with nonstretch group. No significant change was observed in cell proliferation and collagen production in 15% stretch group (Figures 7 and 8).

Cyclic stretching of ACL fibroblast at 1 Hz with magnitude of 5% and 10% showed an increase cell proliferation by 7.1% and 6.1% (*p* < 0.05), respectively. However, at 15% stretch cell proliferation decreased by 7.0% (*p* < 0.05). There was no significant change in collagen production at 5%, 10%, and 15% stretch group (Figures 7 and 8).

### 3.4. Radial Stretch for MSCs

**Figure 9:**
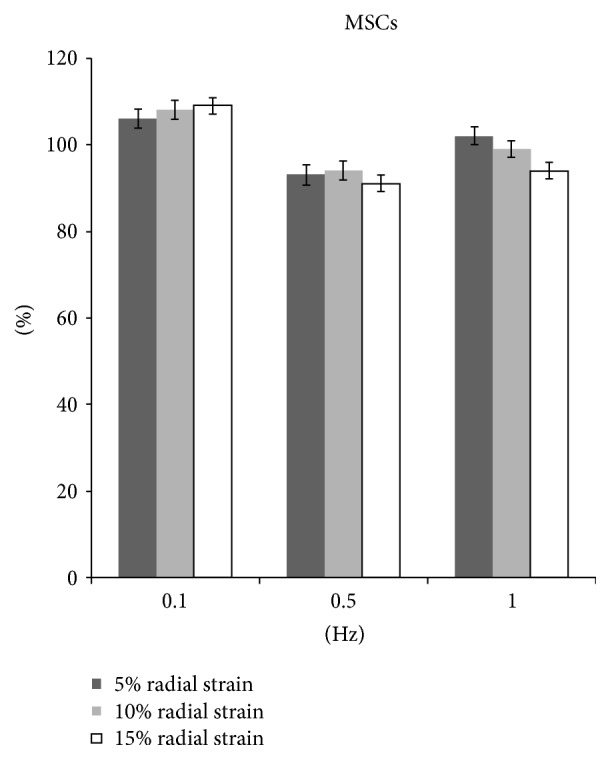
The proliferation of MSCs after radial stretch stimulation.

**Figure 10:**
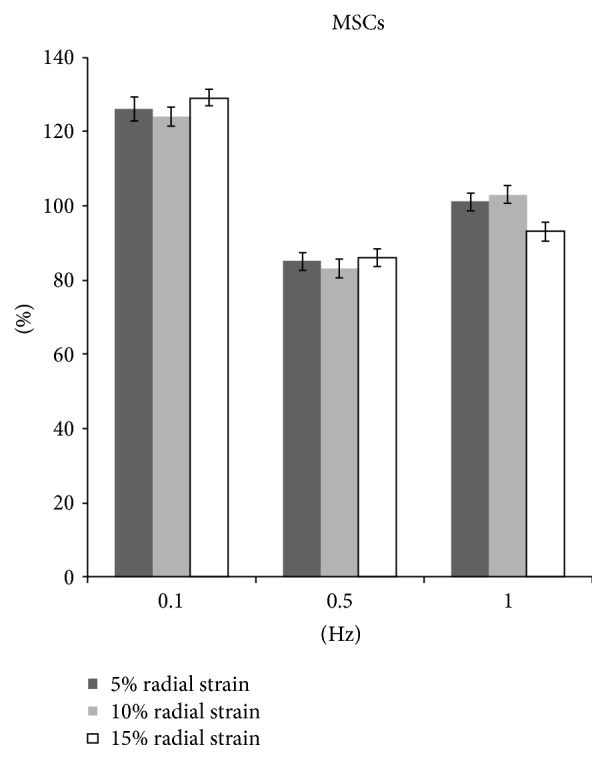
The collagen production of MSCs after radial stretch stimulation.

In comparison with nonstretched group, the MSCs proliferation increased significantly by 6%, 8%, and 9% in 5%, 10%, and 15% radial strain groups, respectively, at 0.1 Hz, 8 hrs/day for 5 days (*p* < 0.05). The amounts of collagen production in all stretching groups were significantly higher than those of control group (Figures 9 and 10).

At 0.5 Hz, the proliferation decreased significantly by 7.0%, 6%, and 9% in 5%, 10%, and 15% strain groups, respectively (*p* < 0.05). Correspondingly, the collagen production also decreased by 15.0%, 16.9%, and 14.0% (*p* < 0.05) (Figures 9 and 10).

At 1.0 Hz, the cell proliferation and collagen production showed no significant difference in 5% stretch and 10% stretch groups. However, at 15% stretch the cell proliferation decreased by 6.0% and collagen production decreased by 6.9% (*p* < 0.05) (Figures 9 and 10).

## 4. Discussion

Ligament is a strong, dense structure made of connective tissue. It connects bone to bone across the joint to keep the dynamic and stable movement. The ACL is one of the most important four strong ligaments connecting the bones of knee joint. The function of ACL is to provide stability to knee and minimize stress across the knee joint. However, it has a poor self-regenerative capacity due to ligament's low cellularity and vascularity. Therefore, it is important to determine the effects of mechanical loading on ACL fibroblast in order to better understand ACL mechanobiology as well as pathophysiology. In addition, the tissue-engineered ligament has been extensively studied in recent years as an alternative graft in preclinical study. Mesenchymal stem cells (MSCs) are among the most promising and suitable stem cell types for ligament tissue engineering. The microenvironment of ACL not only contains biochemical factors but also exerts hemodynamic forces, such as shear stress and cyclic strain, which may influence the differentiation of MSCs [18]. Although many studies investigated the influence of cyclic mechanical stimulation on graft incorporation and cellular differentiation, few literatures have characterized the optimal parameter. In current study, using an in vitro system (Flexcell) that can control the magnitude and frequency of the stretching, the proliferation and collagen production of fibroblast and MSCs were compared to explore the optimal strain condition.

Appropriate mechanical loads at physiological levels would positively influence the expression of ECM and therefore the mechanisms of tendon regeneration. However, while excessive mechanical loading caused anabolic changes in tendons, it also induced differentiation of tendon stem cells into nontenocytes, which may lead to the development of degenerative tendinopathy frequently seen in clinical settings [19]. The mechanical strain used in current study ranged from 5% to 15% elongation, which was within the physiological range experienced by human tendons, given that tendons can elongate by 12–15% [20]. When fibroblasts were uniaxially stretched, the optimal frequency for proliferation and collagen production was 0.5 Hz (Figures 3 and 4). ACL fibroblasts showed an increase in either proliferation or collagen production when they are stretched at different strains (5%, 10%, and 15%).

15% uniaxial strain at 0.5 Hz and 10% uniaxial strain at 1 Hz both stimulated fibroblast proliferation and collagen production. The results indicated that as the frequency increased, lower magnitude of stretch is more favorable for cell proliferation and collagen production. Collagen type I, collagen type III, decorin, and tenascin-C are fundamental proteins in the ECM of tendons [21]. Lohberger et al. [22] stimulated human rotator cuff fibroblast using Flexcell tension system with 10% elongation and a frequency of 0.5 Hz. The total soluble collagen was measured in cell culture supernatants. Cyclic strain significantly increased the collagen production on days 7 and 14. The expression of tenascin-C and scleraxis increased significantly in the mechanically stimulated groups at both time points. There results were correlated with our findings in current study. Uniaxial strain at 0.1 Hz is the least favorable for fibroblast proliferation and collagen production. The cells showed a decrease proliferation and collagen production when they are stretched at 0.1 Hz at different strains (5%, 10%, and 15%) (Table 1).

In contrast to uniaxial strain, 0.5 Hz was least favorable for cell proliferation. Radial strains (5% and 15%) at 0.5 Hz did not have significant effect on cell proliferation. The 10% radial strain showed negative effect and decreased cell proliferation. The strains (5% and 10%) at 1 Hz and 15% strain at 0.1 Hz all stimulated cell proliferation. Interestingly, the collagen production under these conditions showed no significant difference compared to that of nonstretched group. Although the strains (5% and 10%) at 0.1 Hz and 5% strain at 0.5 Hz had no effect on cell proliferation, the cells under these conditions showed significantly increased collagen production (Table 1).

For MSCs under uniaxial stretch condition, 0.5 Hz is favorable for cell proliferation and collagen production. Different strains (5%, 10%, and 15%) all showed positive effects. In addition, 10% strain at 1.0 Hz also upregulated cell proliferation and collagen synthesis. Interestingly, for radial stretch groups, MSCs showed an increase in both proliferation and collagen production when they are stretched at 0.1 Hz at different strains (5%, 10%, and 15%) (Table 2).

In summary, uniaxial stretch (15% at 0.5 Hz; 10% at 1.0 Hz) showed positive effects on fibroblast. The uniaxial strains (5%, 10%, and 15%) at 0.5 Hz and 10% strain at 1.0 Hz showed positive effects on MSCs. Radial strain did not have significant effect on fibroblast. On the contrary, all radial strains (5%, 10%, and 15%) at 0.1 Hz had positive effects on MSCs.

## 5. Conclusion

This study suggested that exposing fibroblasts and MSCs to uniaxial or radial strains promoted cell proliferation and collagen production. The fibroblasts and MSCs had their own appropriate mechanical stimulatory parameters. These specific parameters had great parental application in cell expansion to fabricate tissue engineering products.

## Figures and Tables

**Table 1:** Effects of strains at various frequencies on fibroblasts.

Function	Uniaxial stretch									Radial stretch								
	0.1 Hz			0.5 Hz			1.0 Hz			0.1 Hz			0.5 Hz			1.0 Hz		
	5%	10%	15%	5%	10%	15%	5%	10%	15%	5%	10%	15%	5%	10%	15%	5%	10%	15%
	(strain)			(strain)			(strain)			(strain)			(strain)			(strain)		
Proliferation	↓	↓	↓	↑	↓	↑	↓	↑	↓	**—**	**—**	**↑**	**—**	**↓**	**—**	**↑**	**↑**	**↓**
Collagen	↓	↓	↓	↓	↑	↑	↓	↑	↓	**↑**	**↑**	**—**	**↑**	**↓**	**—**	**—**	**—**	**↓**

“↑”: increase; “↓”: decrease; “—”: no difference (*p* < 0.05).

**Table 2:** Effects of strains at various frequencies on MSCs.

Function	Uniaxial stretch									Radial stretch								
	0.1 Hz			0.5 Hz			1.0 Hz			0.1 Hz			0.5 Hz			1.0 Hz		
	5%	10%	15%	5%	10%	15%	5%	10%	15%	5%	10%	15%	5%	10%	15%	5%	10%	15%
	(strain)			(strain)			(strain)			(strain)			(strain)			(strain)		
Proliferation	**↓**	**↓**	**↓**	**↑**	**↑**	**↑**	**↓**	**↑**	**↓**	**↑**	**↑**	**↑**	**↓**	**↓**	**↓**	**—**	**—**	**↓**
Collagen	**↓**	**↓**	**↓**	**↑**	**↑**	**↑**	**↓**	**↑**	**↓**	**↑**	**↑**	**↑**	**↓**	**↓**	**↓**	**—**	**—**	**↓**

“↑”: increase; “↓”: decrease; “—”: no difference (*p* < 0.05).
